# Thyroid Cancer in Ecuador, a 16 years population-based analysis (2001–2016)

**DOI:** 10.1186/s12885-019-5485-8

**Published:** 2019-04-02

**Authors:** Jorge Salazar-Vega, Esteban Ortiz-Prado, Paola Solis-Pazmino, Lenin Gómez-Barreno, Katherine Simbaña-Rivera, Aquiles R. Henriquez-Trujillo, Juan P. Brito, Theofilos Toulkeridis, Marco Coral-Almeida

**Affiliations:** 1 OneHealth Research Group, Faculty of Medicine, Universidad de las Americas, Calle de los Colimes y Avenida De los Granados, 170137 Quito, Ecuador; 2 Endocrinology Department, Hospital Eugenio Espejo, Quito, Ecuador; 3 Department of Cell Biology, Physiology and Immunology, Universidad de Barcelona, Barcelona, Spain; 4 Faculty of Medicine, Universidad Central del Ecuador, Quito, Ecuador; 5 Division of Endocrinology, Diabetes, Metabolism and Nutrition, Department of Medicine and the Knowledge and Evaluation Research Unit, Mayo Clinic, Rochester, MN USA; 6 Escuela Superior Politecnica del Ejercito, Sangolqui, Ecuador

**Keywords:** Thyroid Cancer, Epidemiology, Ecuador, Latin America, DALY, YYL

## Abstract

**Background:**

Thyroid cancer is the most frequent endocrine neoplasia worldwide. Information from Andean countries is scarce. In Ecuador there is no reports available of the epidemiology of this type of cancer. The aim of this study is to present the epidemiology and the burden of disease of thyroid cancer.

**Methods:**

This is a cross-sectional population-based analysis of thyroid cancer epidemiology in Ecuador from 2001 to 2016. The variables studied were the overall mortality rate, socio-demographics characteristics of the hospitalized patients, geographical trends and the burden of thyroid cancer in Ecuador. All the data was obtained from the official records reported by the Ministry of Public Health’s and retrieved from the public databases of the Vital Statistics Deaths and Births Databases and the National Institute of Census and Statistics (INEC).

**Results:**

In Ecuador, over a period of 16 years from 2001 to 2016 a total of 23,632 hospital admissions were reported, which caused 1539 deaths due thyroid cancer. Data demonstrated an annual mean of 1477 cases, which caused 96 deaths per year in average. The annual incidence fluctuated from 3 in 2001 to 22 in 2016 per 100,000 inhabitants. Women were 5 times more likely than men to have thyroid cancer. The average length of stay for both sexes were 4 days. The mortality attributable to thyroid cancer represent less than 0.3% of all cancer deaths.

**Conclusion:**

Ecuador has one of the highest rates of thyroid cancer in Latin America, ranking first among women in Latin America. Although this cancer is frequent, mortality rate is relatively low. As this is the first national report of thyroid cancer in the country, a further analysis of the pathological variants and the grading of this neoplasia is needed.

## Background

A rapid increase in the incidence of thyroid cancer has been reported in several countries across the world showing significant geographic differences. South Korea, Italy and United States experienced relatively high incidence of thyroid cancer, while some Northern European countries have observed low incidence and minimal increase [1–3]. The underlying causes of this geographic variation remain unknown but difference in health care systems, access to care, cancer screening practices have been proposed as possible reasons for these discrepancies [1].

One geographic area where thyroid cancer epidemiology has been underreported is South America. A few countries such as Brazil (1.8/100,000), Colombia (2.1/100,000) or Chile (7.8/100,000) have reported data about their incidence of thyroid cancer, however, the majority of Latin-American countries such as Ecuador, Peru, Bolivia or Paraguay have not reported thyroid cancer trends, incidences or any epidemiological data in the last decade [4–7]. Understanding different trends and epidemiological variability among Latin American countries will offer more information to help us understand the reasons behind the increasing rates of thyroid cancer worldwide.

The purpose of this study is to describe the incidence and mortality patterns of thyroid cancer in Ecuador from 2001 to 2016, as well as the overall economic impact of thyroid cancer in the country.

## Methodology

### Study population

This is a national-wide cross-sectional design including all the reported cases of thyroid cancer (C-73) in Ecuador in a 16 years period (2001–2016). All the information comes from the public repositories of mortality and hospital discharges obtained from the National Institute of Census and Statistics (INEC).

### Data sources

Information was retrieved from the hospital discharge database in the last 16 years of available data. The website http://www.ecuadorencifras.gob.ec/estadisticas-de-camas-y-egresos-hospitalarios-bases-de-datos/ contains full set of records in a yearly manner.

Thyroid cancer (C-73) was analyze by year with a resolution that included date of discharge by month, cantons (224) and provinces (24) of residence as well as the institutions where treatment was offered. The hospital discharge and the mortality databases contain individual information for demographic characteristics, data on length of stay and mortality, however, the tumor, nodes and metastasis staging (TNM) information was not available. For the burden of disease analysis, the TNM grading was assigned accordingly to the proportion of TNM cancer stage described in previous reports from the country’s National Registry of Cancer published by the National Society of Fighting against Cancer. All data was analyzed by sex, age, marital status, ethnic background, province and canton of residency, educational attainment, and days of hospitalization in the last 16 years.

### Burden of disease

The impact of thyroid cancer was quantify in disability adjusted life years (DALYs), following previously described methods [8]. DALY are the sum of years lived with disability (YLD) and years of life lost due to premature mortality (YLL) [9]. All the cases of thyroid cancer registered as C-73 were included. YLDs were calculated from the hospital discharges databases as the product of the number of incident cases of thyroid cancer, times the disability weight attributed to the disease in the year of diagnosis. Disability weights, described by the Global Burden of Disease 2013 study [10], were assigned accordingly to the proportion of TNM cancer stage described in previous reports from the country’s National Registry of Cancer published by the National Society of Fighting against Cancer (SOLCA) [11]. We used an average disability weight of 0.288 corresponding to diagnosis and primary treatment for stages I to III, and a disability weight of 0.451 for metastatic stage IV [10]. YLLs were estimated as the product of the number of deaths registered due to thyroid cancer and the residual life expectancy at the age of death. Life expectancy was set in 80 years for man and 82.5 for women according to the Coale Demeny model life table system.

### Data analysis

The incidence and mortality rates were age-standardized using the 2010 national population census [12]. The number of cases and deaths were summarized as absolutes numbers and relative frequencies (%). The mortality rate was calculated using the annual population at risk by ethnicity, sex, age group and the geographic location of the incidence. The calculations were completed using the IBM SPSS statistics version 24.0. Citation and retrieval of references were performed using the Zotero Open Source Software version 4.0.11. Spatial analysis was performed using the software QGIS 2.8 and the DALY calculations were performed using STATA software v14. All the graphs and maps were elaborated by the authors.

## Results

### Ecuador

From 2001 to 2016, 23,632 cases of thyroid cancer were diagnosed in Ecuador. The mean age at diagnosis was 49 years (±15 years), 80% were women, with no formal education (45%), mainly diagnosed in urban areas (90%), and treated under public health care system (64%) followed by private for profit (18%), and the private for nonprofit (13%). Thyroid cancer was more frequently reported among mestizos (85%), whites (7%), indigenous (7%) and Afro-Americans (1%).

### Incidence, prevalence and mortality

**Fig. 1:**
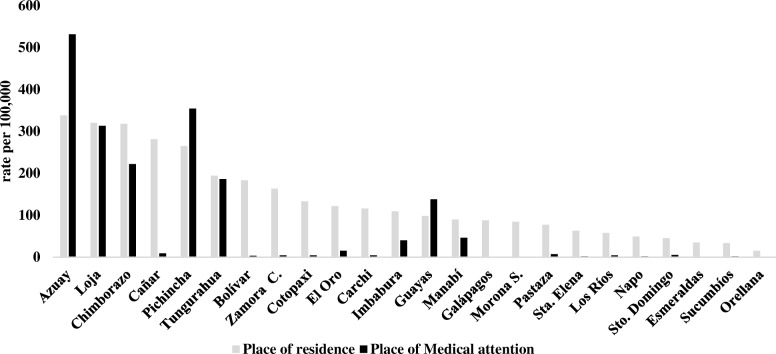
Prevalence rate per 100,000 inhabitants among provinces of residence and provinces where medical attention was given from 2001 to 2016

The annual incidence fluctuated from 3 in 2001 to 22 in 2016 per 100,000 inhabitants, being the annual average prevalence lower in men with 3.5 cases per 100,000 versus 16.6 cases per 100,000 in women (2001–2016). The prevalence rates present geographic variation ranging from 3 cases per 100,000 individuals in Orellana to 338 cases per 100,000 individuals in Azuay (Fig. 1).

**Fig. 2:**
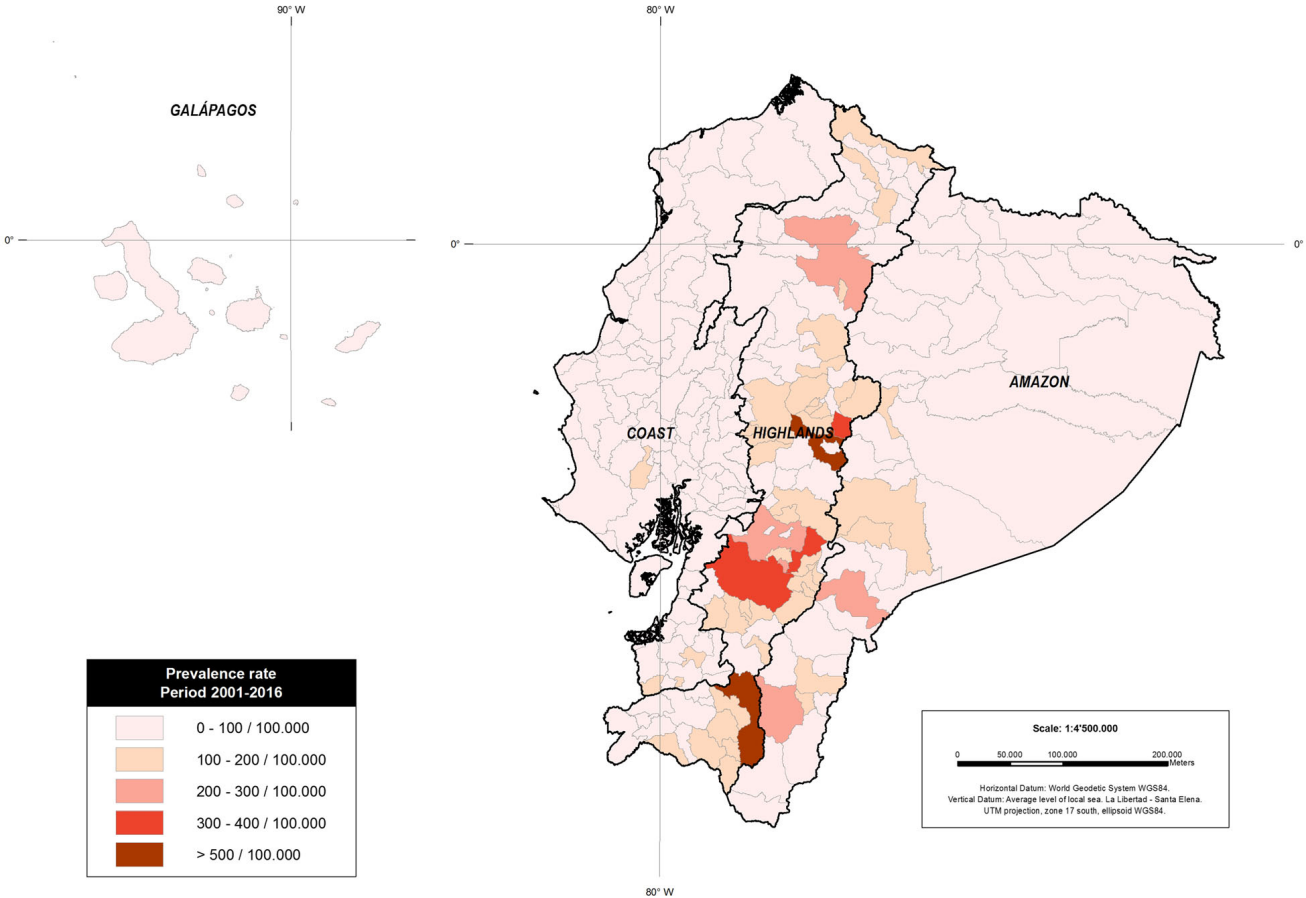
prevalence rates of thyroid cancer according to Cantons from 2001 to 2016

When analyzed by cantons, the canton with highest prevalence rates was Riobamba with 435 cases per 100,000 individuals while the lowest prevalence rates was 2.3 cases per 100,000 individuals in San Lorenzo, Esmeraldas (Fig. 2).

The differences in prevalence rate change from region to region. The highlands have a prevalence rate of 122/100,000, followed by the Amazonia with 47/100,000 and the Coastal region (Including the Galapagos islands) a prevalence of 46/100,000 (Fig. 2).

**Fig. 3:**
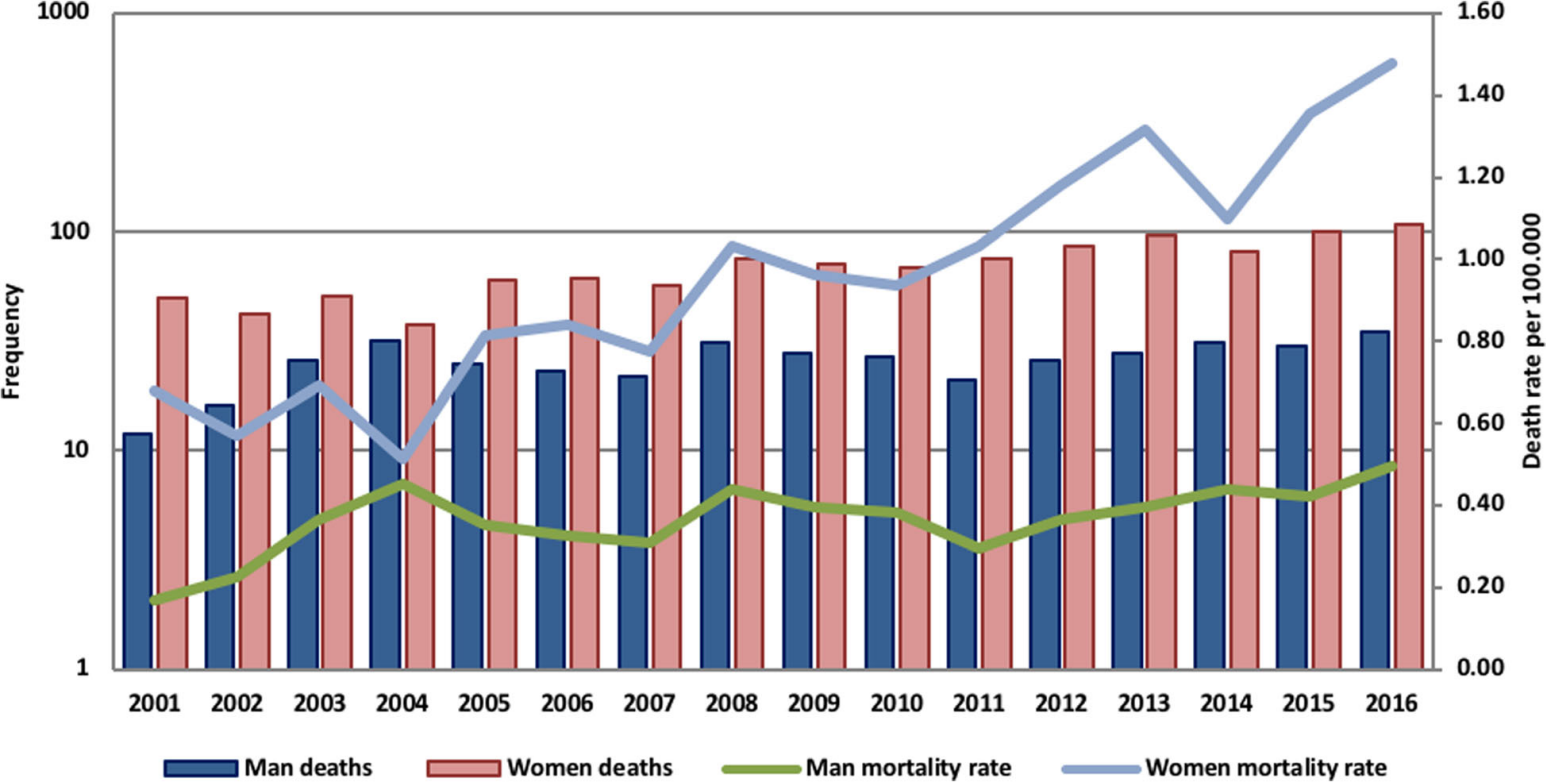
The annual deaths rate per 100,000) of Thyroid cancer in Ecuador from 2001 to 2016 in men and women

**Fig. 4:**
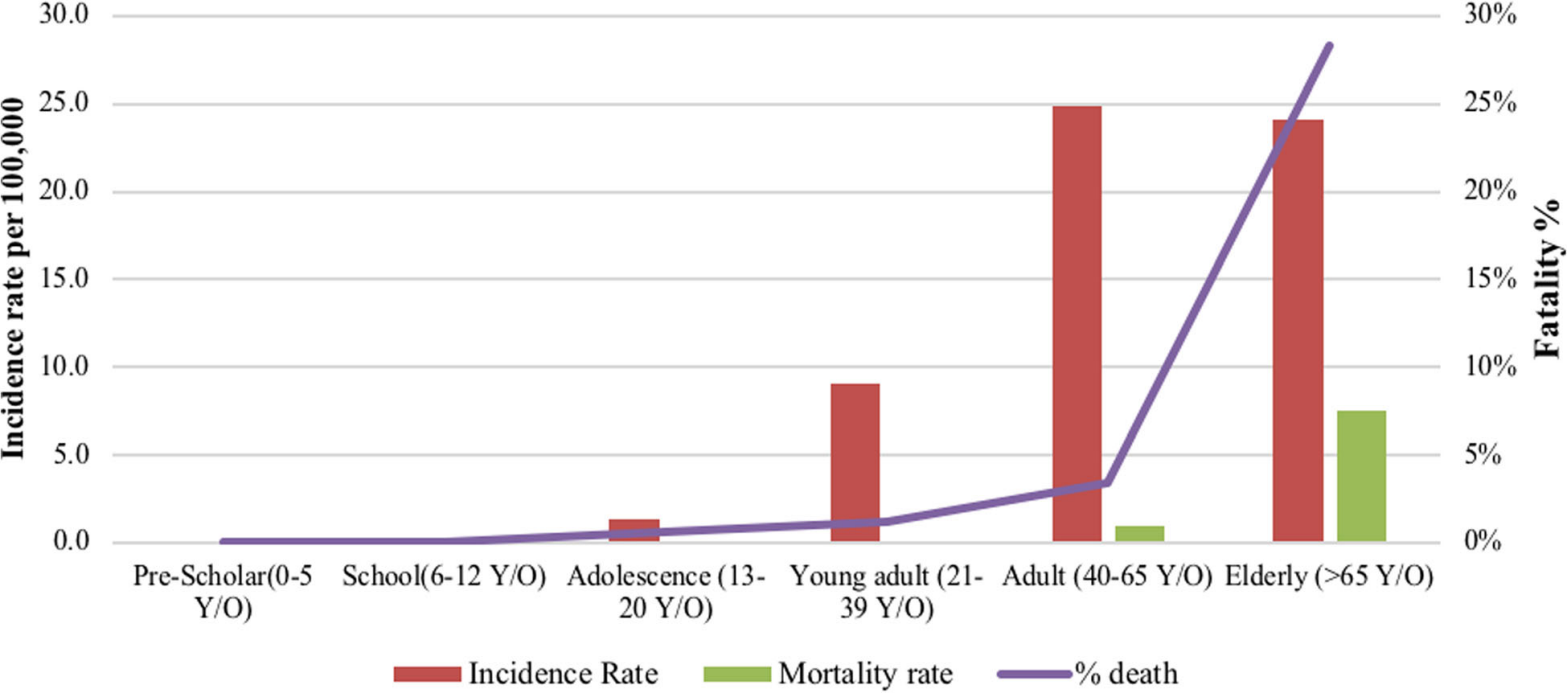
Number of cases and fatalities by age group and the overall mortality from 2001 to 2016

Thyroid cancer mortality according to death rates at all ages per 100,000 was found to be 0.36 deaths in men and 0.95 deaths in women per 100,000 with a female to male mortality of 2:1 (Figs. 3 and 4).

When analyzed by age groups, children and adolescents, mortality is 0%, slightly increasing up to 0.1% in young adults (21 to 39 years old) reaching the highest mortality among elderly people with thyroid cancer, with morality incidence of 7 per 100,000 (figure 4100).

### Trends

**Fig. 5:**
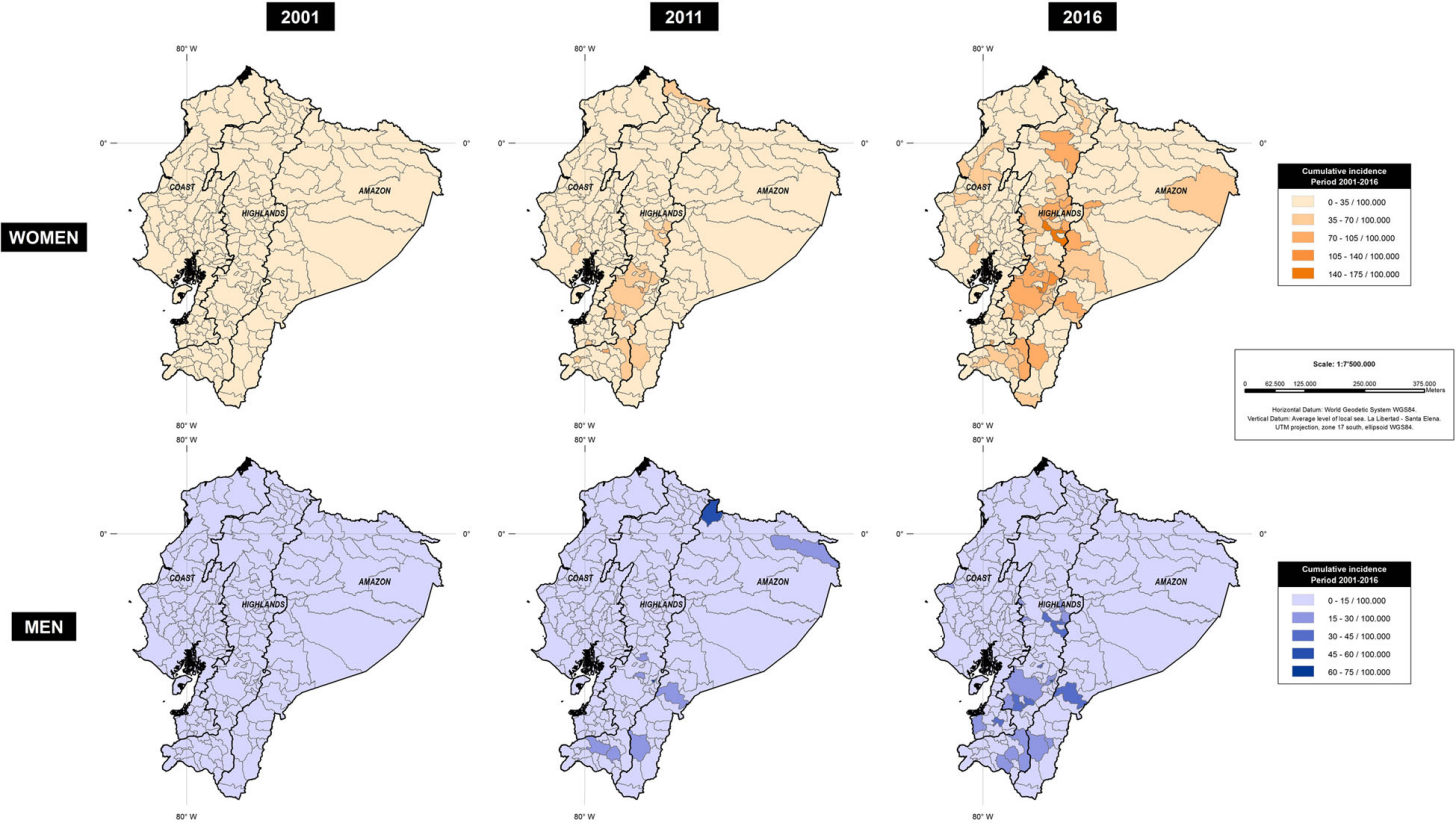
Average incidence (rate per 100,000) of Thyroid cancer in Ecuador in 2001, 2011 and 2016

2001 to 2016 the incidence of thyroid cancer increased from 3 to 22 per 100,000 individuals, during the same time thyroid cancer mortality increased from 0.48 to 0.87 per 100,000 individuals in the overall 16 years period. The incidence trend analysis stratified by gender revealed increasing rates in women, from 4.3 to 42.2 cases per 100,000 individuals, while in men from 0.97 to 8.4 cases per 100,000 individuals (Fig. 5).

### DALYs and YLLs analysis

During the period 2001–2016 in Ecuador there were 23,632 patients who were admitted to the hospital with diagnosis of thyroid cancer. In this time period 1539 deaths were attributed to thyroid cancer. These deaths are equivalent to 26,202 years of life lost due to premature mortality (Table 1).

**Table 1:** Burden of disease estimation of Thyroid cancer (ICD-10: C-73) in Ecuador from 2001 to 2016

Year	YLL	YLD	DALY	DALY per 100 k pop.
2001	1173	118	1291	10.1
2002	1229	130	1359	10.4
2003	1547	152	1699	12.8
2004	1225	171	1396	10.3
2005	1352	205	1557	11.3
2006	1443	236	1679	12.0
2007	1393	293	1685	11.9
2008	1692	343	2035	14.1
2009	1633	337	1971	13.4
2010	1499	411	1910	12.7
2011	1664	500	2164	14.2
2012	1779	594	2373	15.3
2013	2017	736	2754	17.5
2014	1810	872	2682	16.7
2015	2116	998	3115	19.1
2016	2630	1134	3764	22.8

*YLL* years life lost due to mortality, *YLD* years lived with disability, *DALY* disability adjusted life years

## Discussion

Thyroid cancer is a relatively common malignancy in Ecuador. The incidence and mortality rates had a significantly variation from 2001 to 2016. During this period, the number of cases increased almost 20 times. Our report demonstrated that in the study population the ratio women/men is 5:1.

According to global reports, the incidence and mortality of thyroid cancer in Ecuador (both male and female) exceeds the rest of countries in Latin America, and is among the highest in the world [5, 13] (Table 2). Remarkably, in older adults (> 65 years) the mortality is greater than 25%. We hypothesized that it might be due to comorbidities more frequents in this age.

**Table 2:** Age-standardized incidence and mortality rates (per 100,000) in Latin-America

Country	Period	Incidence			Mortality		
		F	M	F:M	F	M	F:M
Argentina	2003–2007	5.5	1.4	4	0.4	0.3	1.3
Brazil	2003–2007	14.4	3.4	4.2	0.4	0.2	2
Chile	2003–2007	8.2	1.4	5.7	0.6	0.4	1.5
Colombia	2003–2007	10.7	2.5	4.2	0.8	0.4	2
Costa Rica	2003–2007	12.6	2.1	6.1	0.5	0.4	1.3
Cuba	2004–2007	7.7	1.5	5.2	0.4	0.3	1.3
Ecuador	2003–2007	16	3.5	5.0	0.9	0.5	2.6
Mexico	2006–2010	4.9	1.2	4	0.9	0.5	1.8
Peru	2001–2005	7.5	1.7	4.4	0.7	0.3	2.3
Uruguay	2005–2007	6.8	1.6	4.2	0.4	0.4	1

When we refer to the group of children and adolescents, we have found that they are still relatively unaffected by thyroid cancer, representing less than 2% of the overall incidence countrywide [2, 14].

Over these 16 years, the significant increase of the thyroid cancer had an evident influence in the disability adjusted life years with a rise of 12.7 DALY per 100.000 depending mainly on younger adults (Table 1) [15, 16].

Part of the debate encloses some of the arguments that explain a higher incidence of thyroid cancer, in the last decade. Over-diagnosis in fact due to new technologies that permit early diagnosis of a greater number of small tumours that are usually clinically undetectable, but evident by radiological and diagnostic techniques like CT scan or ultrasound respectively [13, 17–20]. In recent years in Ecuador, a better healthcare and improved (both public and private) access might be one of the reasons related with a possible over utilization of diagnostic tools [21, 22].

The influence of other factors such as the use of radioactive drugs (i.e. iodine-123 or iodine-131) in hyperthyroid patients has never been explored in the country, however recently published studies have established a possible link between the use of radioactive iodine and some forms of thyroid cancer [23, 24].

In Ecuador like in many Latin American countries with similar standard of care and comparable health systems, information about screening test that resulted in false positives is not available. In despite of this, countries in other regions have reported that massive screening is related to a higher rate of thyroid cancer with no direct impact on the overall mortality [1, 25–27].

Our results demonstrate that certain ethnic backgrounds have higher incidence of thyroid cancer, than others. For instance, in Ecuador the racial thyroid cancer’s distribution is greater among mestizo population, maybe due to the fact that mestizos are the greater population in the country, having similar mortality rates among them [12]. There is some data reporting that thyroid cancer’s survival rates might be different among populations, in example, in the United States of America, non-Hispanic whites are less affected than the Hispanic and African-American populations [28, 29].

It is relevant to point out that 45% of patients had poor educational attainment even though more than 90% of the cases were diagnoses among urban dwellers. These socioeconomic conditions are similar to those reported in other studies and might influenced the detection patterns (and therefore incidence) of thyroid cancer in the country [30–33].

The majority of patients with thyroid cancer, reside within the highland’s region. The provinces of Azuay, Loja and Chimborazo have the highest number of affected. The canton with the highest incidence was Riobamba in the province of Chimborazo. The results of our study show that, on average, the prevalence rates are higher in the mountainous regions of Ecuador (averaged elevation 2358 m) compared to those of the Amazon region (averaged elevation 731 m) and the coast (averaged elevation 93 m). The association between elevation and thyroid cancer was not profoundly explored in our investigation, however, some reports suggest that a link between extreme geographies and thyroid cancer might have some biological plausibility [34–36].

The role of iodine supplementation and the risk of developing hypothyroidism, goitre and cretinism was explored in Ecuador and Peru during the 60’s [37, 38]. The quality of the soils and the lack of iodine was established as one of the main reasons of the greater incidence of goitre among high altitude dwellers when compared to their coastal peers [37]. An efficient and worldwide salt iodization campaign reduced the trends of goitre and hypothyroidism, nevertheless the quality of the soils, neither the average intake of iodine has been ever explored in Ecuador.

It could be speculated that after decades of high iodine intake, a paradoxical effect might have occurred in some parts of Ecuador, leading to higher risk of developing thyroid tumours [39–41]. Reports from the past have shown that overstimulating the thyroid gland with iodine-rich diets and high iodine intake might be related with an increased risk of developing thyroid tumours in animal models [42, 43]. In humans, some of the studies available reporting a possible association between iodine intake and thyroid cancer have led to controversial results [39, 40, 44–46].

### Limitations

The main limitation of this type of analysis is the lack of individual’s data concerning the tumor stratification and the risk factors attributed to developing thyroid cancer. Another major limitation is that the current study cannot separate thyroid cancer by histological subtype. The use of nationwide secondary data could lead to information bias, particularly relevant for the validity of the confirmatory diagnosis of thyroid cancer and the lack of country guidelines for accurate diagnosis.

## Conclusion

Describing the local epidemiology of thyroid cancer is an important step in order to improve health access and to promote evidence-based health public policies. This report is the first in Ecuador analyzing the epidemiology and burden of diseases of thyroid cancer with a cantonal resolution. The results confirm that Ecuador has the highest rates in Latin America of this type of neoplasia, which follows a continues increment in the incidence of thyroid cancer, without changing mortality in the same rate as the diagnosis does. A further analysis is needed in order to investigate the risk factors associated with higher risk of developing this type of cancer in Ecuador, especially that attributed to high altitude exposure, soil composition and iodine intake.

## Abbreviations

C-73: Thyroid cancer
DALY: Disability adjusted life years
GCP: Good clinical practices
INEC: National Institute of Census and Statistics
TNM: Tumor, Nodes and Metastasis Staging System
YLD: years lived with disability
YLL: Years of life lost
